# Effects of meniran (*Phyllanthus niruri* L.) administration on leukocyte profile of broiler chickens infected with *Mycoplasma gallisepticum*

**DOI:** 10.14202/vetworld.2018.834-839

**Published:** 2018-06-22

**Authors:** Sri Hidanah, Emy Koestanti Sabdoningrum, Retno Sri Wahjuni, Sri Chusniati

**Affiliations:** 1 Department of Animal Husbandry, Jl. Mulyorejo, Kampus C Unair, Surabaya, Indonesia, 60115; 2 Department of Basic Veterinary Medicine, Jl. Mulyorejo, Kampus C Unair, Surabaya, Indonesia, 60115; 3 Department of Microbiology, Faculty of Veterinary Medicine, Universitas Airlangga, Jl. Mulyorejo, Kampus C Unair, Surabaya, Indonesia, 60115

**Keywords:** chicken, leukocyte, *Mycoplasma gallisepticum*, *Phyllanthus niruri* L

## Abstract

**Aim:**

This study aimed to evaluate the effects of Meniran extract (*Phyllanthus niruri* L.) administration on leukocyte profile of broiler chickens infected with *Mycoplasma gallisepticum*.

**Materials and Methods:**

Thirty broiler chickens, 21 days old were divided into five treatment groups. P0 (−): Chickens without any treatment; P0 (+), P1, P2, and P3: Chickens were infected with *M. gallisepticum* 10^8^ cells/ml/animal orally, then given no treatment, Meniran extract 60%, 62.5%, and 65% orally at a dose of 1 ml/kg body weight, respectively. The treatment of Meniran extract was given for 7 days.

**Results:**

Leukocyte count with the lowest number showed in Group P0 (−) and Group P3 (p>0.05). Increased number of basophils was found in Group P0 (+), Group P1, and Group P2. The highest number of heterophils was found in Group P0 (+) and was significantly different from Group P0 to P3 (p<0.05). The same pattern was also seen in the number of lymphocytes in all treatment groups. The number of monocytes showed no significant difference between all treatment groups (p>0.05).

**Discussion:**

Increased the number of leukocytes is often observed in inflammation due to general infections, trauma, or toxicity. Shifting in the number of heterophile or lymphocytes, an increase in the number of monocytes, basophils, and eosinophils may also be associated with various infectious or inflammatory conditions. Heterophils play a role as an antibacterial defense through several effective mechanisms. When infections and inflammation occur, the heterophils will increase to phagocytosis microbe.

**Conclusion:**

It can be concluded that Meniran extract (*P. niruri* L.) at a dose of 65% can decrease the total number of leukocytes in broilers infected with *M. gallisepticum*.

## Introduction

Endemic disease that attacks broiler chicken farms is considered as one of the obstacles of broiler chicken business since it will cause huge economic losses. One of the diseases that can lead to huge economic losses for broiler chicken farms is respiratory disease [1]. In the respiratory disease, bacterial infection appears to play the greatest role. One of the bacteria capable of causing respiratory disease in broiler chickens is *Mycoplasma gallisepticum*, also known as the cause of chronic respiratory disease (CRD) [2].

*M. gallisepticum* is not an invasive bacterium, but it can spread through the gaps of the respiratory organ cells due to their small size and through blood capillaries [3] by hemorrhaging erythrocytes [4]. It will then trigger changes in the number of leukocytes in the blood due to bacterial infection in the body. In other words, increased leukocytes can be considered as a physiological response to protect the body from invading microorganisms, especially neutrophils [5].

CRDs have commonly been treated using antibiotics to inhibit protein syntheses, such as tiamulin and tylosin. Those antibiotics can damage the cell wall. However, certain antibiotics such as penicillin and its derivatives will not be useful since *M. gallisepticum* bacteria do not have a cell wall. Besides, continuous administration of antibiotics can also cause broiler chickens to be resistant to the drugs and leave a dangerous residue for them [6].

On the other hand, Meniran (*Phyllanthus niruri* L.) is a medicinal plant commonly found in Indonesia. The plant contains various chemical compounds such as lignans, tannins, polyphenols, alkaloids, flavonoids, terpenoids, and steroids [7]. Some of those chemical compounds, such as alkaloids, flavonoids, saponins, and tannins, are thought to have antimicrobial activity [8]. In addition to antimicrobial activity, Meniran extract also can serve as an immunomodulator that will enhance the immune system components as well as improve the disrupted immune system function [9]. Thus, it is necessary to conduct research on effects of Meniran extract (*P. niruri* L.) administration on leukocyte profile of broiler chickens infected with *M. gallisepticum*.

## Materials and Methods

### Ethical approval

The entire research was conducted appropriately following the ethics in using experimental animals and has been approved by the ethics committee of the Faculty of Veterinary Medicine, Universitas Airlangga.

### Research location and time

This research was conducted at several places in the Veterinary Medical Faculty of Universitas Airlangga, such as the animal cage unit, the Veterinary Clinical Pathology Laboratory of the Basic Medicine Veterinary Department, the Bacteriology and Microbiology Laboratory of the Microbiology Department, the Molecular Biology Laboratory, and the Pharmacology Laboratory of Basic Medical Science Department.

### Research materials

Research materials used in this research were commercial chicken feed (PT Charoen Pokphand) and vitamins regularly administered during the research, Meniran extract (*P. niruri* L.), 96% ethanol and Carboxymethyl Cellulose (CMC Na), *M. gallisepticum* isolates, 70% alcohol, wright stain solution, as well as oil immersion.

### Research tools

Research tools used were cage, feeding place, drinking place, gloves, mask, ethylenediaminetetraacetic acid (EDTA) tube, glass cover, tissue, microscope, glass object, paint shelf, blood cell counter, hematology analyzer, test tube, filter tool, rotary evaporator, oven, measuring cup, Bunsen, pipette, Erlenmeyer tube, basin, two 50-watt incandescent lamps, ND vaccine (new castle disease), disinfectant soap, cage sized 60 cm×50 cm×40 cm, and battery cage sized 40 cm×40 cm×37 cm.

### Treatment

Chickens aged 21 days were proved to be infected with avian *M. gallisepticum* before the experiment. Chickens showed respiratory symptoms at 3 days after infection. The presumptive diagnosis was based on the occurrence of typical signs (mucus discharge from the mouth and nostrils and increased respiratory rate) together with differential diagnosis (isolation and identification of the causative organisms), and final diagnosis was based on serological tests.

Thirty broiler chickens were divided into five treatment groups as follows:

P0 (−): A negative control group of chickens without any treatment
P0 (+): A positive control group of broilers orally infected *M. gallisepticum* bacteria with a concentration of 10^8^ cells/ml/animal without the administration of Meniran extract.
P1: A treatment group of broiler chickens infected with *M. gallisepticum* bacteria with a concentration of 10^8^ cells/ml/animal, then given 60% Meniran extract at a dose of 1 ml/kg body weight (BW).
P2: A treatment group of broiler chickens infected with *M. gallisepticum* bacteria with a concentration of 10^8^ cells/ml/animal, then given 62.5% Meniran extract at a dose of 1 ml/kg BW.
P3: A treatment group of broiler chickens infected with *M. gallisepticum* bacteria with a concentration of 10^8^ cells/ml/animal, then given 65% Meniran extract at a dose of 1 ml/kg BW.
Meniran extract was given for 7 days orally using probe.

### Preparation of ethanol extract of Meniran (*P. niruri L.*)

First, Meniran was aerated under the shade. Second, Meniran that has been dried was ground into powder. Third, 1 kg of Meniran powder was extracted with maceration technique by immersing it in 5 L of 96% ethanol solution for 3×24 h. Fourth, it was stirred twice, morning and evening. In total, the maceration process was conducted 3 times. Fifth, the results of the immersion in the form of filtrates were then filtered for subsequent evaporation using a rotary evaporator resulting in Meniran extract solution [10].

### Multiplication of *M. gallisepticum*

Mycoplasma broth isolates were obtained from Balai Besar Veteriner Wates. Agar culture media for bacteria not yet filled were prepared. Multiplication of *M. gallisepticum* bacteria was then performed by striking the agar culture media with a sterile inoculating loop/transfer loop. Afterward, the agar medias were put into jars containing wet cotton since the bacteria grow in an anaerobic state. The jars then were put into an incubator at 37°C for 3-21 days to grow bacteria on those agar culture medias [11].

### Preparation of *M. gallisepticum* suspension

Bacterial suspension was made by preparing the suspension of *Mycoplasma gallisepticum* bacteria. Next, the suspension of *M. gallisepticum* bacteria was centrifuged for 10 min at 5000 rpm. The top of the suspension of *M. gallisepticum* bacteria in the centrifuge tube was then removed to obtain its filtrate remaining at the bottom of the tube. Afterward, the filtrate was mixed physiologically with NaCl using a vortex at 3000 rpm, and then centrifuged as many as 3 times [12].

The infectious dose of *M. gallisepticum* bacteria was determined using McFarland standard # 1. It means that 3×10^8^ cells were diluted to obtain a dose of 10^8^ cells/ml/animal. The dilution of the infectious dose of *M. gallisepticum* bacteria was started by adding 9 ml of NaCl solution into the tubes. The tubes that had been filled the NaCl solution then were added to *M. gallisepticum* bacterial suspension. Next, the bacterial suspension was added to make their turbidity equal to the McFarland standard #1 tube with the number of bacterial colonies of 3×10^8^ colony-forming unit (CFU). After their turbidity appeared the same, the suspension of 3×10^8^ CFU bacteria was diluted to 10^8^. The dilution was performed with a ratio of 1-2, i.e., 1 ml of McFarland standard #1 solution together with the number of bacterial colonies (3×10^8^) was added into 2 ml of physiological NaCl solution [13].

### Blood sampling

Blood sampling was taken through brown brachialis vein as much as 1 cc. Blood samples examined were given anticoagulant EDTA. The blood samples with a dose of 1 mg/1 ml then were put into the EDTA tube. Next, the total number of leukocytes and the number of each type of leukocytes were examined at the Veterinary Clinical Pathology Laboratory of the Basic Medicine Veterinary Department of the Veterinary Faculty of Universitas Airlangga.

### Statistical analysis

Data about the total number of leukocytes and the number of each type of leukocytes then were statistically analyzed with ANOVA test and then continued with Duncan multiple stability test using SPSS 21 for windows with a significance level of 95% (p≤0.05).

## Results and Discussion

Results of the leukocyte count analysis showed that there was no significant difference between Group P0 (−) and Group P3 (p>0.05). However, both were significantly different from Group P0 (+), Group P1, and Group P2 (p<0.05). The mean and standard deviations of Group P0 (−), Group P0 (+), Group P1, Group P2, and Group P3 were, respectively, 20.440±1.430, 38.800±5.833, 34.400±4.615, 33.258±3.014, and 25.400±3.130 (Table-1).

**Table-1:** The total number of leukocytes in the broiler chickens infected with *M. gallisepticum* bacteria after the treatments.

Treatment	Total number of leukocytes (X±SD) (10^3^/mm^3^)
P0-	20.440^a^±1.430
P0+	38.800^c^±5.833
P1	34.400^b^±4.615
P2	33.258^b^±3.014
P3	25.400^a^±3.130

*Different superscripts in the same column indicated a significant difference (p<0.05). *M. gallisepticum*=*Mycoplasma gallisepticum,* SD=Standard deviation

Increased leukocyte production suggests that the body responds to infection, drug reactions, or immune system disorders [14]. Difference in the leukocyte profile was found between the two treatment groups of those broiler chickens infected *M. gallisepticum* bacteria treated with 60 and 62.5% Meniran extracts (Group P1 and Group P2).

On the other hand, the total number of leukocytes decreased in Group P1, Group P2, and Group P3. This condition indicates that the inflammatory process due to bacterial infection had stopped. This is due to the ability of Meniran plant extract in killing bacteria. Meniran extract is known to contain tannins, saponins, and alkaloids that have antibacterial activity [15]. Antibacterial agents have various ways of killing bacterial growth, one of which is by destroying the structure of the bacterial cells, resulting in the death of bacterial cells [16].

In general, an increase in the number of leukocytes in peripheral blood is often observed in stressful conditions triggered by inflammation due to general infections, trauma, toxicity, neoplasms, etc., whereas decline may be an indication of swelling or chronic infection [17,18]. However, changes in specific cell populations can be seen in a variety of different conditions.

Furthermore, the calculation results of the number of each type of leukocytes in the treatment groups indicated varying results. The number of eosinophils in Group P0 (−) was not significantly different from that in Group P1 and Group P2 (p>0.05). There was a significant difference in the number of eosinophils between Group P0 (+) and Group P3 (p<0.05). Similarly, there was also a significant difference in the number of eosinophils between Group P0 (+) and Group P3 (p<0.05). The lowest number of eosinophils was found in Group P3 about 500±111, while the highest one was found in Group P0 (+) about 1,337±156. However, they are still within the normal range of eosinophil count in chickens, about 0-1,000 cells, except Group P0 (+) with the number of eosinophils above the normal one.

Unlike the number of eosinophils, the number of basophils was rarely found in the blood samples of those broiler chickens. There was not a significant difference in the number of basophils between Group P0 (−) and Group P3 (p>0.05). Similarly, there was also not a significant difference in the number of basophils between Group P1 and Group P2. Nevertheless, the number of basophils in all four treatment groups was significantly different from Group P0 (+) (p<0.05). The mean number of basophils in Group P0 (−), Group P0 (+), Group P1, Group P2, and Group P3 was, respectively, 10±1, 110±25, 262±37, 230±48, and 11±2. Increased number of basophils was found in Group P0 (+), Group P1, and Group P2.

In addition, the number of heterophils in Group P0 (−) was not significantly different from Group P3. Similarly, the number of heterophile in Group P1 was not significantly different from Group P2 (p>0.05). However, the other three treatment groups were significantly different from Group P0 (+) (p<0.05). The number of heterophils in Group P0 (−) and Group P3 was still at normal one, while the number of heterophils in Group P0 (+), Group P1, and Group P2 was above the normal one. The same pattern was also seen in the number of lymphocytes in all treatment groups.

Unlike the number of other leukocyte components, the number of monocytes showed a different pattern. The number of monocytes showed no significant difference between all treatment groups (p>0.05) (Table-2).

**Table-2:** The number of each type of leukocytes in broiler chickens infected with *M. gallisepticum* bacteria after the treatment

Treatment	Eosinophils (10^3^/mm3)	Basophils (10^3^/mm3)	Heterophils (10^3^/mm3)	Lymphocytes (10^3^/mm3)	Monocytes (10^3^/mm3)
P0 (-)	736^b^±151	10^a^±1	5.150^a^±290	13.040^a^±269	1.500^a^±117
P0 (+)	1.337^c^±156	110^b^±25	10.100^c^±200	24.595^c^±696	2.658^a^±299
P1	873^b^±85	262^c^±37	8.990^b^±226	21.157^b^±348	3.118^a^±122
P2	700^b^±158	230^c^±48	8.529^b^±247	20.992^b^±210	2.807^a^±938
P3	500^a^±111	11^a^±2	5.387^a^±155	17.411^a^±112	2.001^a^±251

* Different superscripts on the same line showed a significant difference (p<0.05). *M. gallisepticum*=*Mycoplasma gallisepticum*

Moreover, eosinophilia is rarely found in chickens. However, if it occurs, it can be attributed to parasitism (mites, intestines, parasites, and parasites with tissue migration) [19]. Basophils have an ability to release histamine and control reactions of allergics and antigens invading the body [20]. In chickens, the high number of basophils in the blood indicates that they are in abnormal conditions, such as stress due to sufficiently hot air or facing pathogen infection [21]. Meanwhile, the low number of basophils indicates that chickens are in healthier conditions [22]. The number of heterophils and lymphocytes showed the same pattern in this study. The higher number of heterophils and lymphocytes was found in Group P0 (+), Group P1, and Group P2 than in the other treatment groups, i.e. P0 (−) and P3.

Increased heterophils can be found in bacterial, fungal, and parasitic infections, inflammation, stress, toxicity, traumatic conditions, as well as leukemia [17,23]. Certain infectious conditions, such as bacterial infections or extraordinary viral diseases in hematopoietic cells, can cause a decrease in the number of heterophils [24]. Increased lymphocyte count may be associated with inflammatory effects due to *M. gallisepticum* infection, chronic infection or inflammatory conditions that can be attributed to certain viral infections [18,23], while a decrease in the number of lymphocytes can occur under stress conditions [24,25]. Chronic antigenic stimulation that can lead to the extensive circulation of lymphocytes since the primary function of the lymphocyte is involved in the immunological response, the formation of humoral antibodies mediated by immunocompetent cells.

Depending on a variety of conditions, shifting in the number of heterophile or lymphocytes may produce changes in the ratio of heterophils to lymphocytes. An increase in the number of monocytes, basophils, and eosinophils may also be associated with various infectious or inflammatory conditions [24].

The decrease or increase of leukocyte count, furthermore, will always be accompanied by a change in leukocyte count profile. Heterophilia accompanied by lymphocytosis in Group P0 (+) may be due to the consequent need of bacterially infected tissue to heterophils and lymphocytes since both are effective against microbial in particular bacteria or can also be caused by high levels of stress [26].

In addition, heterophils play a role as an antibacterial defense through several effective mechanisms, i.e., chemotaxis (an ability of heterophile to attract to the site of infection and inflammation) and as phagocytosis, i.e., an ability of heterophile to eat and destroy microbes. When infections and inflammation occur, the heterophils will increase to phagocytosis microbe. At the same time, the bone marrow is stimulated to release more heterophile cells in the blood, and there is leukocytosis characterized by increased young leukocytes [27].

In Group P1 (treated with Meniran extract at a dose of 60%), Group P2 (treated with Meniran extract at a dose of 62.5%), and Group P3 (treated with Meniran extract at a dose of 65%), the number of heterophile significantly decreased due to the function of tannin having toxic properties to bacterial cell membranes by inhibiting certain enzymes that will damage microbial or bacterial cells [28]. The antibacterial activity of this tannin can also inhibit bacterial cell wall synthesis, then will change the permeability of cell membrane or active transport through the cell membrane, as well as inhibit protein synthesis and nucleic acid synthesis, thus, the process of bacterial growth will be damaged [29].

Besides, neutrophils have strong potency in quantity, chemotaxis, and phagocytosis [30]. The potential of such neutrophils can be enhanced by the provision of immunomodulators, such as *P. niruri* L. A previous research has proven that *P. niruri* L. is capable of enhancing neutrophil chemotaxis [31], affecting the number of neutrophils and their speed in reaching the inflammatory area thus increasing their effectiveness in eliminating bacteria.

*P. niruri* L. is an immunomodulator that stimulates the immune response, but can also regulate excessive immune responses. Much of the biological and chemical content is found in *P. niruri* L. [31,32], and some have anti-inflammatory effects. *P. niruri* L. also demonstrates an ability to inhibit the production of nuclear factor-κB (NFκB) *in vitro*. NFκB is needed to induce interleukin (IL)-8 as a major mediator for neutrophils *Phyllanthus* may also inhibit the induction of IL1β and interferon gamma (IFNγ) in whole blood and the reduction *in vivo* of tumor necrosis factor α (TNFα). IL-1β, IFNγ, and TNFα have an important role in an acute inflammatory process including activating neutrophils [33,34].

Based on the results of an *in vitro* research, the administration of *P. niruri* L. extract is known to affect non-specific immune responses in the forms of increased phagocytosis, macrophage chemotaxis, neutrophil chemotaxis, 42 natural killer cell cytotoxicity, and complement activation [35]. The administration of *P. niruri* L. extract also can attack specific immune responses by increasing the proliferation of T lymphocytes, increasing the secretion of TNFα, IFNγ, and IL-4, as well as decreasing IL-2 and IL-10. Besides, the administration of *P. niruri* L. extract can trigger humoral immunity by increasing the production of immunoglobulin M as well as immunoglobulin G [36].

Finally, *P. niruri* L. is thought to have anti-inflammatory effects. *P. niruri* L. demonstrates some abilities to inhibit nitric oxide 31 (NO) and prostaglandin E-2, reduce endotoxin-induced NO synthase and cyclooxygenase, as well as inhibit NFκB production *in vitro*. *P. niruri* L. is also known to inhibit the induction of IL-1β, IL-10, and IFNγ in whole blood as well as the reduction *in vivo* of TNFα. A previous research on the use of *P. niruri* L. in animals even reveals that it can increase the activity of various antioxidant enzymes, such as superoxide dismutase, catalase, glutathione-S-transferase, glutathione peroxidase, and glutathione reductase, and in both the blood and tissues reduced during radiotherapy, thus reducing cellular damage due to radiotherapy [37].

## Conclusion

It can be concluded that *P. niruri* L. extract at a dose of 65% can decrease the total number of leukocytes in broilers infected with *M. gallisepticum* bacteria into nearly its normal one.

## Authors’ Contributions

SH, EKS, and RSW carried out the main research works, EKS and SC performed the statistical analysis of the data in the experiments, and all of the authors approved the final manuscript.
